# Axial sesamoiditis in the horse: A review

**DOI:** 10.4102/jsava.v89i0.1544

**Published:** 2018-03-29

**Authors:** Christelle le Roux, Ann Carstens

**Affiliations:** 1 Department of Companion Animal Clinical Studies, University of Pretoria, South Africa; 2 Department of Small Animal Clinical Studies, University of Pretoria, South Africa

## Abstract

Axial sesamoiditis or osteitis of the proximal sesamoid bones (PSBs) in the horse is described as a rare condition. The cause remains unknown and speculative, with vascular, infectious and traumatic aetiologies implicated. It is specifically associated with injury of the palmar or plantar ligament (PL), also known as the intersesamoidean ligament. Imaging findings are generally rewarding, and radiological changes are typical, if not pathognomonic, for the condition. Lesions consist of bone lysis at the apical to mid-body axial margins of the PSBs, with variable degrees of joint effusion. Radiographic technique warrants careful attention to make a diagnosis, and exposure factors may need to be adjusted. Perineural, intra-articular and intra-thecal anaesthesia does not seem to provide consistent improvement of lameness in these cases, with literature reporting inconsistent findings. Ultrasonographic findings include digital flexor sheath effusion, loss of the normal fibre structure of the PL at its attachment to the PSBs, abnormal echogenicity or change in thickness of the PL, and irregular hyperechoic cortical margins of the axial margins of the PSBs. Scintigraphy, computed tomography and magnetic resonance imaging, although not necessary to make a diagnosis, may add valuable information regarding the location and extent of lesions. The prognosis remains guarded to poor for return to athletic function. The focus of this article is a comprehensive review of the proposed aetiopathogenesis of the condition, the prognosis and a summary of the literature findings with focus on the notable diagnostic imaging features, including radiography, ultrasonography, scintigraphy, computed tomography and magnetic resonance imaging.

## Introduction

**FIGURE 1:**
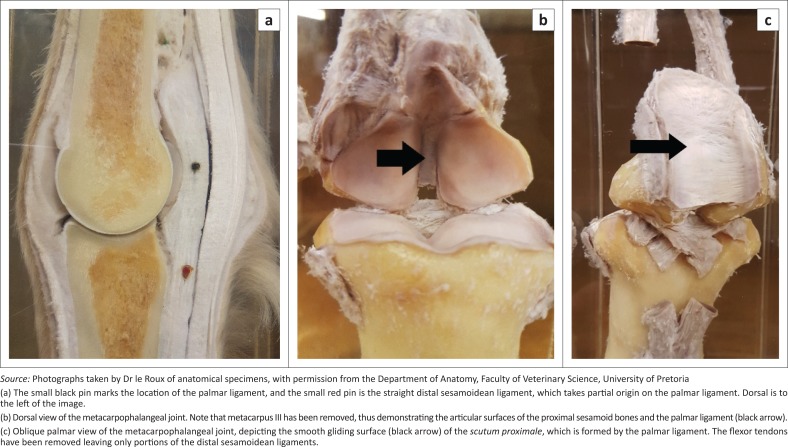
(a) Midline sagittal, (b) dorsal and (c) oblique palmar photographs of anatomical specimens of the metacarpophalangeal joint of a horse.

Axial sesamoiditis or osteitis of the proximal sesamoid bones (PSBs) in the horse is described as a rare condition, with approximately 56 cases reported in the literature, with 32 of these cases described in the past 5 years (Brommer et al. 2014; Dabareiner et al. 2001; Formston & Serth 1968; King et al. 2013; Lawrence & Fraser 2013; Sedrish, Burba & Williams 1996; Sherman, Myhre & Heymann 2006; Vanderperren et al. 2014; Wisner et al. 1991). The cause remains unknown and speculative (Sherman et al. 2006). It is considered to be a separate clinical entity from the typical more abaxial presentation of proximal sesamoiditis, as it is specifically associated with injury of the palmar or plantar ligament (PL), also known as the intersesamoidean ligament (Dabareiner et al. 2001; Richardson & Dyson 2011). The PL along with the PSBs forms part of the metacarpophalangeal joint’s (MCPJ) and metatarsophalangeal joint’s (MTPJ) *scutum proximale.* The PL is a thick collagenous structure firmly attached to the PSBs by a layer of fibrocartilage, creating a firm union between the two bones (Denoix, Busoni & Olalla 1997). It provides a smooth gliding surface for the flexor tendons and prevents contact between the palmar and plantar surfaces of metacarpus and metatarsus (MC/MT) III and the tendons, especially in hyperextension of the fetlock joint (Figure 1).

## Proposed aetiopathogenesis

The aetiological factors for axial sesamoiditis have been speculative, with vascular, infectious and traumatic aetiologies implicated. Generally, aetiological factors can be divided into non-septic and septic causes (Dabareiner et al. 2001).

**FIGURE 2:**
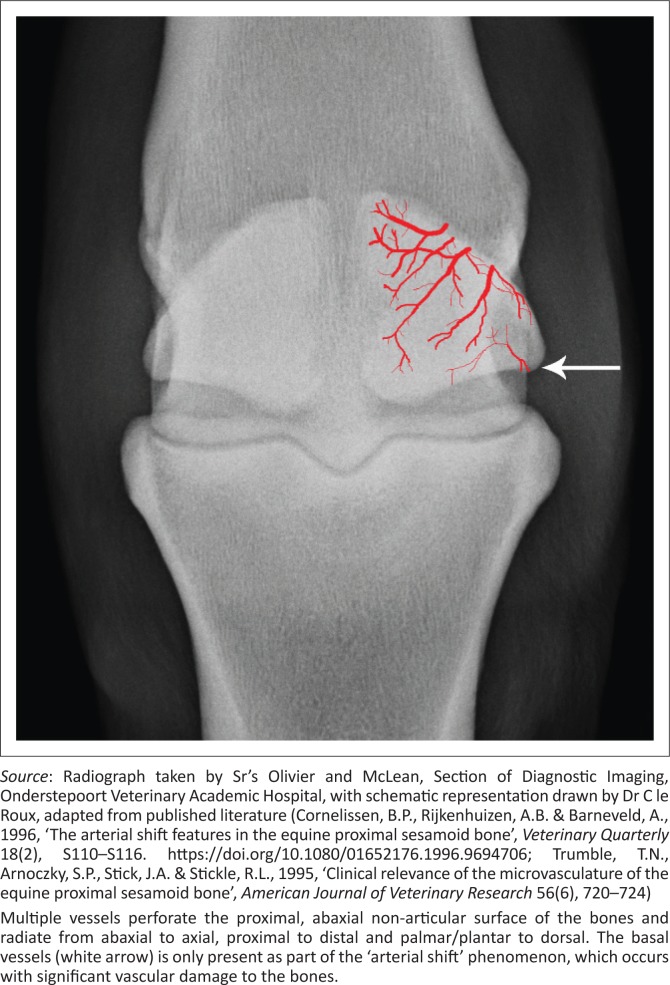
Dorsoproximal-palmarodistal oblique radiographs of the left metatarsophalangeal joint of a 6-year-old Friesian stallion, with a schematic representation of the blood supply to the proximal sesamoid bones superimposed on the image.

Blood supply to the PSBs comes from the medial and lateral palmar or plantar digital arteries, with multiple vessels perforating the proximal, abaxial non-articular surface of the bones and then radiating from abaxial to axial, proximal to distal and palmar or plantar to dorsal within the PSBs (Figure 2) (Barr et al. 2005; Cornelissen, Rijkenhuizen & Barneveld 1996; Trumble et al. 1995). The PSBs are for the most part well vascularised, but the PL and axial aspects of the PSBs are less well-supplied, and when digital arteries are perfused with barium sulphate, the axial borders of the PSBs are perfused last (Sherman et al. 2006). However, the vascular supply of the PSBs is easily damaged with trauma, for example, with fracture of a PSB, and thus, these findings are well correlated with regard to the frequency of non-union fractures of the PSB (Trumble et al. 1995). It is speculated that avulsion of the PL with hyperextension of the MCPJ and MTPJ may cause disruption to the blood supply of the PSB and lead to axial osteolysis (Dabareiner et al. 2001).

Unfortunately, there is still much debate regarding the significance of the poor blood supply, with studies indicating that even with iatrogenic occlusion of the sesamoidean arteries, causing disruption to a large part of the PSBs, lameness was always mild and resolved relatively rapidly. Histopathology and radiographs also demonstrated little major change in these cases. These findings are in contradiction to the often severe chronic and insidious onset of lameness seen in axial sesamoiditis with marked radiographic changes. The chronic nature of axial sesamoiditis affords ample time for collateral circulation to develop, and thus, appreciable bony pathology should not occur (Cornelissen et al. 2002). This collateral circulation of the PSBs is in part thought to be because of an ‘arterial shift’, where a usually insignificant basal sesamoid blood supply takes over after damage to the sesamoidean artery. It is clear that more research needs to be carried out into the importance of blood supply with regard to PL damage and axial sesamoiditis.

Trauma, specifically hyperextension of the MCPJ or MTPJ, with excessive stresses on the PSB and tearing or avulsion of PL, has also been implicated in the pathogenesis of axial sesamoiditis (Dabareiner et al. 2001; Sherman et al. 2006; Vanderperren et al. 2014). Barclay, Foerner and Phillips (1985), in their report on axial sesamoid injuries occurring concurrently with lateral condylar fractures, described two horses with obvious axial sesamoid fractures and two horses with more progressive axial demineralisation. These axially located PSB injuries had a poor outcome for future performance, with persistent lameness. Rotation of the palmar aspect of the metacarpal condyles against a stationary phalanx one, first from medial to lateral and then from lateral to medial during maximal hyperextension, is suggested to put severe strain on the PL and PSBs (Barclay et al. 1985).

The Friesian horse, which has more elastic tendons than had previously been described in Thoroughbreds, has been reported to be a breed more commonly affected by axial sesamoiditis and PL desmitis (Boerma, Back & Sloet van Oldruitenborgh-Oosterbaan 2012; Brommer et al. 2014; Vanderperren et al. 2014). Seven out of 18 horses in the most recent study on axial sesamoiditis were Friesians, and although this may reflect a specific hospital population, breed-associated predisposition could be a valid consideration (Vanderperren et al. 2014). Tendon and ligament laxity may lead to fetlock hyperextension and resultant PL desmitis, because of interbone ligamentous laxity. It has been suggested that many of the problems experienced in this breed (not only related to the musculoskeletal system) are collagen-related and systemic collagen-linked abnormalities play a role (Boerma et al. 2012). These may inadvertently have been preferentially selected when breeding for the typical standards of this breed.

Infectious causes of axial sesamoiditis primarily consist of bacterial septic arthritis, which is associated with more severe lameness and joint effusion. There may also be extension of infection from a septic digital flexor sheath tenosynovitis (Roos 1972), or extension from the primary site of axial sesamoiditis to the digital flexor tendon sheath (DFTS) (Sherman et al. 2006). In the majority of cases of septic axial sesamoiditis, neither a traumatic wound nor other cause of introduction for the infection is ever identified (Formston & Serth 1968; Wisner et al. 1991). Seeding of bacteria following intra-articular injections has also been proposed as a cause of the condition (Bertone 2011).

In Wisner et al.’s (1991) study of seven horses with axial sesamoiditis, three horses had septic tenosynovitis of the DFTS, and two of those horses also had concurrent septic arthritis. Unfortunately, not all horses in this study had arthrocentesis, synoviocentesis of the DFTS or post-mortem studies performed. A study of eight horses by Dabareiner et al. (2001) revealed that three out of eight horses had evidence of sepsis of the fetlock joint and digital sheath.

A single case report describes the catheterisation of the dorsal metatarsal artery for blood pressure monitoring during general anaesthesia, leading to axial sesamoiditis in three horses, with septic thrombosis of the microvasculature of the PSB thought to play a role (Barr et al. 2005). This case differs somewhat from other described cases because of the variation in location of the PSB lesions, with two out of three cases occurring axially.

Intra-articular corticosteroid injections may predispose to fungal sesamoiditis, although the actual frequency of occurrence in horses is unknown (Sherman et al. 2006). Sequestrum formation has also been described following PSB sesamoiditis, and contrary to the rapid healing of sequestrae in long bones of horses that undergo surgical treatment, PSB sequestration results in a prolonged healing period possibly because of its poor blood supply (Dunkerley, Hanson & Humburg 1997).

## Clinical examination

Lameness is usually moderate to severe (grade 3 to 5 out of 5), may be intermittent and is often of variable duration prior to presentation, with reports ranging from a few days to several months (Wisner et al. 1991). Some horses may present acutely, but without history of trauma or penetrating wounds (Dabareiner et al. 2001; Wisner et al. 1991).

In the majority of cases, only one limb is affected (Dabareiner et al. 2001; Formston & Serth 1968; Sedrish et al. 1996; Wisner et al. 1991), with a suspected predisposition for the hindlimbs. Five out of eight horses in a study by Dabareiner et al. (2001) and five out of seven horses in a study by Wisner et al. (1991) had hindlimb involvement. A study of 12 affected Friesian horses demonstrated exclusively hindlimb involvement (Brommer et al. 2014), with 14 out of 18 horses in another recent study demonstrating hindlimb involvement (Vanderperren et al. 2014). Both PSBs of a limb are commonly affected (Wisner et al. 1991). Wisner et al.’s (1991) study found three out of seven horses to have both fetlock joints affected, but only one case could be identified as bilaterally affected on radiographs, whereas the other two required computed tomography (CT) for identification. The authors have seen one case of bilateral hindlimb involvement, and axial sesamoiditis has been reported to develop in the contralateral limb weeks after rehabilitation for the affected limb (Brommer et al. 2014).

Clinical examination may reveal MCPJ or MTPJ or DFTS distension, pain on flexion of the MCPJ or MTPJ, limited extension of the joint and pain on direct palpation of the PSBs (Vanderperren et al. 2014; Wisner et al. 1991). Diffuse cellulitis over the affected joint has also been described (Dabareiner et al. 2001).

Perineural (abaxial sesamoid and low palmar or plantar blocks), intra-articular and intra-thecal (DFTS) anaesthesia do not seem to provide consistent improvement of lameness in these cases, with literature reporting inconsistent findings (Dabareiner et al. 2001; Sedrish et al. 1996; Wisner et al. 1991). It is speculated that variation in communication between the plantar and palmar pouch of the MCPJ or MTPJ with the DFTS may be the reason for this, likely secondary to the PL pathology. It appears that it may be helpful to perform anaesthesia of all three compartments to obtain a diagnosis (Dabareiner et al. 2001).

## Diagnostic imaging findings

Four standard radiographic views of the fetlock are recommended for evaluation of PSBs, including dorsoproximal-palmarodistal oblique (DPr-PaDiO, which best demonstrates the lesions), lateromedial (LM), and two oblique views – dorsomedial-palmaro lateral oblique (DM-PaLO) and dorsolateral-palmaromedial oblique (DL-PaMO) (Butler et al. 2016; Wisner et al. 1991). In the pelvic limb, the term plantaro (Pl) will replace the term palmaro. It is important to increase kilovoltage settings for the dorso-palmar or plantar views in order to visualise the axial borders of the PSBs (Butler et al. 2016), and poor radiographic technique may be a reason for not visualising these lesions. The palmaroproximal-palmarodistal oblique (PaPr-PaDiO) has not received much attention according to the literature, but allows for the evaluation of the palmar, axial and abaxial aspects of the PSBs and may be helpful for evaluating osteolysis of the axial margins of the PSBs (Richard & Alexander 2007). Bony changes may not be apparent when radiographs are first taken, as up to 30% – 60% of mineral content must be lost, and 7–10 days are needed before changes become detectable (Dennis et al. 2010). Therefore, repeating radiographs after this time may be helpful. The condition may be bilateral in the Friesian, and therefore, it may be advisable to at least obtain a DPa or DPl radiographs of the opposite limb for evaluation in this breed (Brommer et al. 2014).

**FIGURE 3:**
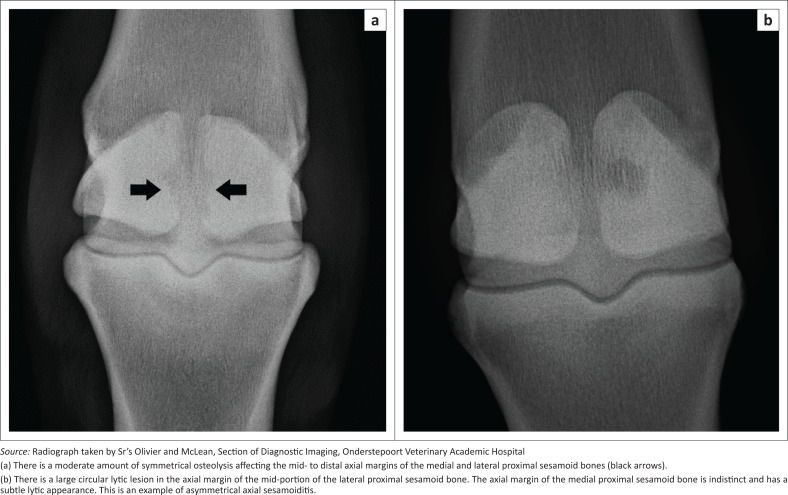
Dorsoproximal-palmarodistal oblique radiographs of the (a) left metatarsophalangeal joint of a 6-year-old Friesian stallion (b) and of the left metacarpophalangeal joint of a 15-year-old warmblood mare. Lateral is to the right of the images.

Radiological findings are typical, if not pathognomonic, for axial sesamoiditis and remain the mainstay for diagnosis of this condition. Lesions consist of bone lysis at the apical to mid-body axial margins of the PSBs, although the entire axial border can be involved (Figure 3). Variable degrees of joint effusion may be present. Lesions may be symmetrical or asymmetrical between the two PSBs, and may either appear cystic or erosive, and are exclusively destructive in nature (Wisner et al. 1991). Sequestrum formation has been described but is uncommon (Dabareiner et al. 2001; Dunkerley et al. 1997). The reader is referred to further radiological examples of the condition elsewhere (Bertone 2011; Butler et al. 2016).

**FIGURE 4:**
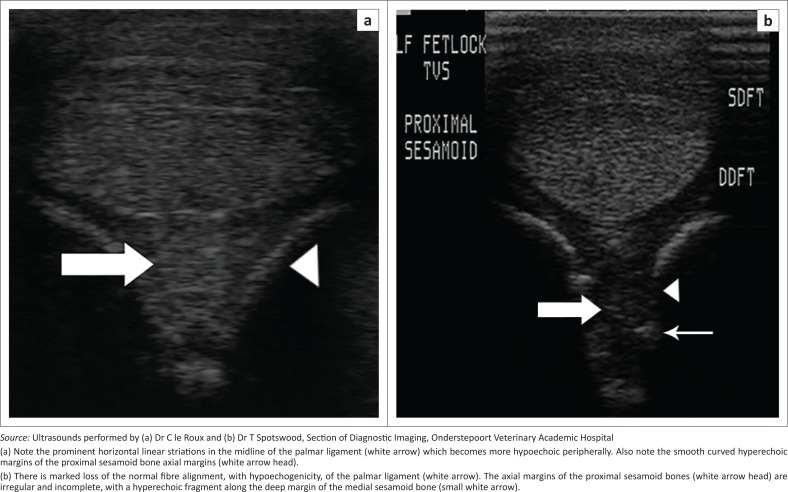
(a) Transverse ultrasound scans performed at the level of the palmar proximal sesamoid bones of the left forelimbs of a normal palmar ligament and sesamoid bones of an adult mixed breed horse (b) and a limb affected by axial sesamoiditis and palmar desmitis in an adult Thoroughbred horse. Lateral is to the left of the images.

Ultrasonography is a valuable tool to visualise the PL, abaxial margins of the PSBs, digital flexor sheath and flexor tendons. The normal PL is approximately 13 mm – 17 mm thick and of moderate echogenicity, with only the sagittal portion having a typical linear hyperechoic structure, similar to other ligaments (Denoix et al. 1997). This is because of the non-linear fibre orientation of the ligament and hence differences in angles of insonation of the ultrasound beam. Abnormalities detected by ultrasound may include DFTS effusion, loss of the normal fibre structure of the PL at its attachment to the PSBs, altered and abnormal echogenicity or change in thickness of the PL, and irregular hyperechoic cortical margins of the axial margins of the PSBs (consistent with enthesopathy) or hyperechoic avulsion fragments (Figure 4) (Dabareiner et al. 2001; Vanderperren & Saunders 2009; Vanderperren et al. 2014). Normal ultrasonographic findings of the MCPJ and MTPJ, as well as abnormalities consistent with axial sesamoiditis, have been further reported in detail (Denoix et al. 1997).

**FIGURE 5:**
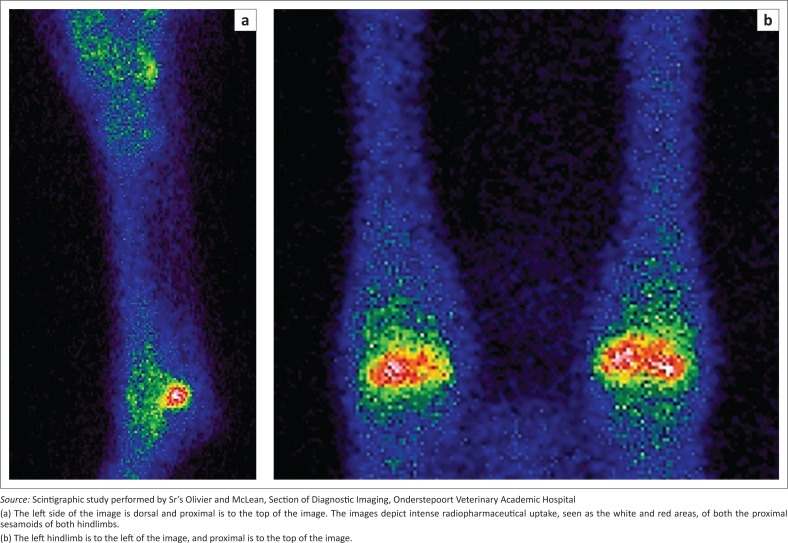
(a) Lateral left distal hindlimb and (b) plantar bilateral distal hindlimb scintigraphy images acquired in the bone phase of a 6-year-old Friesian stallion (same horse as in Figures 2 and 3).

Scintigraphy typically shows an increased radiopharmaceutical (^99m^ technetium-methylene diphosphonate) uptake in the bone phase of the study of most lesions, with the exception of very small or inactive lesions (Figure 5). Although not necessary for diagnosis, it may evaluate the activity and significance of radiographically visualised lesions (Wisner et al. 1991) and may help localise the source lameness (Dabareiner et al. 2001). This may be especially important in cases where older lesions are present, as it has been described that axial lytic lesions may persist for months. Follow-up radiographs obtained in two foals with axial sesamoiditis demonstrated that osteolytic lesions were still well visualised up to 15 months after lameness had resolved (Lawrence & Fraser 2013). Unfortunately, facilities to perform scintigraphy are of limited availability to the private practitioner in South Africa.

Computed tomography has identified lesions of axial sesamoiditis not seen on radiographs and has shown lesions that were identified to be larger than those seen on radiographs (Vanderperren et al. 2014; Wisner et al. 1991). The benefits of CT are because of its ability to depict cross-sectional anatomy without superimposition of structures, its improved contrast resolution, especially for bone, and the ability to reformat an image in any plane (called multiplanar formatting) after acquisition. Intra-arterial contrast CT imaging may reveal contrast enhancement within the PL, consistent with either abnormal blood vessel permeability, disruption of the blood vessels or neovascularisation of damaged tissues (Brommer et al. 2014; Vanderperren et al. 2014). Intra-articular contrast injection has been described as a technique that can demonstrate communication between the MCPJ or MTPJ and the sesamoid lesions or the DFTS (Vanderperren et al. 2014). Disadvantages of CT include the need for specialised facilities and training, general anaesthesia of the horse in many cases and limitation of the size of the patient or body part that can be accommodated by the gantry size (Peterson & Bowman 1988). Unfortunately, equine CT is of limited availability to most private practices in South Africa.

Magnetic resonance imaging (MRI) findings have shown abnormal increased signal intensity within the ligament, and/or high signal intensity within the axial aspects of the sesamoid bones on short-Tau-inversion recovery (STIR) or proton density sequences, consistent with inflammation, necrosis or scar formation (Brommer et al. 2014; King et al. 2013). Although MRI is not required to make a diagnosis of axial sesamoiditis, it is often performed after radiographs have failed to demonstrate a cause of lameness. MRI for equines is currently not available in South Africa.

## Arthrocentesis

Cytological findings in axial sesamoiditis are unfortunately quite variable, with inflammatory (non-degenerative neutrophilic fluid), infectious (degenerative neutrophilic fluid) and even normal findings described (Wisner et al. 1991). Often, aerobic and anaerobic cultures are negative; however, *Pseudomonas* spp., *Enterobacter* spp. and have been cultured from affected joint and DFTS aspirates (Dabareiner et al. 2001).

## Arthroscopy

Arthroscopy of the palmar MCPJ or plantar MTPJ joint pouch reveals PL damage, consisting of fraying, discolouration and separation from the PSBs, and osteomalacia and osteochondral fragmentation of the axial borders of the PSBs (Dabareiner et al. 2001). There may be fraying of the dorsal surface of the deep digital flexor tendon because of contact between it and the PSBs, with severe PL damage. The PL may be completely disrupted or torn (Brommer et al. 2014).

## Histopathology and post-mortem findings

Histopathological changes of the PL may range from acute to chronic, which is consistent with the duration of clinical signs (Wisner et al. 1991). Acute changes included thrombosis of the blood supply to the PL, necrosis and neutrophilic infiltration of the PL, and fibrinopurulent osteomyelitis and abscessation of the PSBs. Chronic changes consisted of fibroblastic tissue replacing the PL collagenous matrix as well as the osseous lesions, all consistent with chronic degenerative inflammation (Dabareiner et al. 2001; Wisner et al. 1991). Histopathological findings do not appear to differ between horses affected by sepsis and those showing non-septic inflammatory changes (Dabareiner et al. 2001).

Post-mortem findings usually consist of hyperaemia and defects or fissuring of the PL gliding surface and brown discolouration of the collagenous substance of the ligament (Wisner et al. 1991). There may be abnormal effusion of the DFTS, with hyperaemic synovium and fibrin tags, and there may be fibrinopurulent arthritis.

## Outcome and prognosis

Debate exists on the long-term outcome and prognosis of axial sesamoiditis. Evaluation of outcome was hampered in the past by several horses undergoing euthanasia without any treatment (Wisner et al. 1991). Several studies indicate a poor prognosis for return to full work (Richardson & Dyson 2011; Wisner et al. 1991), whereas others indicate a more favourable prognosis for the aseptic form treated with arthroscopic or tenoscopic debridement (Dabareiner et al. 2001). Five out of six horses in Dabareiner et al.’s (2001) study that returned to work had the aseptic form, and all had arthroscopic and/or tenoscopic debridement as part of their treatment protocol. One horse remained mildly lame as a result of development of osteoarthrosis of the affected fetlock joint. In Wisner’s (Wisner et al. 1991) study, none of the seven horses returned to work; however, none had surgical intervention. Only three of the seven horses underwent treatment, which consisted of systemic antibiotics and MCPJ or MTPJ joint lavage. It appears that medical treatment alone carries a poor prognosis.

Septic forms generally carry a poor prognosis, and the general consensus appears to be that of a poor to guarded prognosis (Dunkerley et al. 1997; Sedrish et al. 1996; Wisner et al. 1991). Lawrence and Fraser (2013) reported findings on two foals with septic axial sesamoiditis in which a successful outcome was obtained with the use of oral doxycycline, which is not an antibiotic administered previously for this condition. Unfortunately, the effect of axial sesamoiditis on sesamoid strength and future athletic performance is not known despite resolution in the two foals, as they were not old enough to enter training at the time of the reported findings (Lawrence & Fraser 2013).

The Friesian breed has also been reported to carry an especially poor prognosis (Brommer et al. 2014), with return to light riding being the best possible outcome. Treatment options in a retrospective study of 12 Friesian horses consisted additionally of osteoclast inhibitors (sodium tiludronate), intra-articular medication into the MTPJ (triamcinolone acetonide or platelet rich plasma), orthopaedic shoeing or radial pressure wave therapy; no one treatment appeared to have a better outcome compared to others (Brommer et al. 2014). The authors have seen one case of a 6-year-old Friesian stallion that made a reasonable recovery and was in light work 4 months after arthroscopic treatment.

## Conclusion

Axial sesamoiditis is a clinical entity in which the imaging findings are well documented and rewarding, but treatment and long-term outcome are often disappointing, and a poor prognosis can be expected in the majority of cases.
